# Effects of active localization and vascular preservation of inferior parathyroid glands in central neck dissection for papillary thyroid carcinoma

**DOI:** 10.1186/s12957-020-01867-y

**Published:** 2020-05-13

**Authors:** Dawei Hou, Haie Xu, Bing Yuan, Jianhui Liu, Yibing Lu, Ming Liu, Zhuyin Qian

**Affiliations:** 1 Department of General Surgery, The Second Affiliated Hospital of Nanjing Medical University, 121 Jiangjiayuan Street, Gulou District, Nanjing, 210011 NO China; 2 The School of Public Health, Nanjing Medical University, Nanjing, 211166 China; 3 Department of Endocrinology, The Second Affiliated Hospital of Nanjing Medical University, Nanjing, 210011 China; 4 Department of Medical Information and Data, The Second Affiliated Hospital of Nanjing Medical University, Nanjing, 210011 China

**Keywords:** Thyroid cancer, Papillary, Parathyroid glands, Neck dissection, Lymph node excision, Blood vessel preservation

## Abstract

**Background:**

The purpose of present study is to assess the effects of active localization and vascular preservation of inferior parathyroid glands in central neck dissection (CND) for papillary thyroid carcinoma (PTC).

**Methods:**

A classification of IPGs according to their location and vascular features was developed, and, based on this classification, a CND procedure was designed, and IPGs and their vascular were actively localized and strategically preserved. A total of 197 patients with PTC who underwent a total thyroidectomy and concomitant CND were enrolled. Eighty-nine patients with traditional meticulous fascia dissection were allocated to group A, and 108 patients with active location and vascular preservation of IPGs were allocated to group B. Those with inferior parathyroid glands auto-transplantation in each group were assigned as group At (18) and group Bt (12). Variables including serum intact parathyroid hormone (PTH), total calcium, the incidence of transient, and permanent hypoparathyroidism were studied.

**Results:**

Compared with group A, serum intact PTH (*P* < 0.001) and total calcium levels (*P* < 0.05) in group B significantly improved on the first postoperative day, and the incidence of transient hypoparathyroidism significantly dropped in group B (*P* < 0.001). A total of 170 patients in the two groups had complete follow-up data. The incidence of permanent hypoparathyroidism significantly decreased in group B, from 8.8% to 1.0% (*P* = 0.017). However, there were no significant differences in all variables between group Bt and group At.

**Conclusion:**

Active location and vascular preservation of inferior parathyroid glands effectively protected the function of IPGs in CND for PTC.

## Background

Thyroid carcinoma is a common malignant disease of the thyroid. The incidence of thyroid carcinoma has steadily increased worldwide over the past few decades [1]. Papillary thyroid carcinoma (PTC) is the most common pathologic type of thyroid carcinoma and accounts for about 80% of all thyroid carcinoma [2]. Although PTC commonly exhibits indolent oncological behavior, it has the preference of regional lymph node metastasis [2]. Lymph nodes of the central neck compartment, including the paratracheal, pre-tracheal, and pre-laryngeal lymph nodes, are the most common locations involved [3]. It had been proven that 40-60% of patients with PTC showed lymph node metastasis at the time of the primary operation [3, 4].

Due to PTC’s commonly seen regional lymph node metastasis, central neck dissection (CND) has been advocated by American Thyroid Association (ATA) in patients with advanced PTC (T3, T4 stage) and clinical lymph node metastasis [5]. However, the routine performance of prophylactic CND is controversial due to the hypoparathyroidism that it may cause [5–7]. A multiple-centered study demonstrated that 16.2% of patients that underwent total thyroidectomy and concomitant bilateral central neck dissection developed permanent hypoparathyroidism [8]. Because permanent hypoparathyroidism severely impairs the quality of life of patients and considerably increases their medication costs [9–11], it is meaningful to explore how to protect the parathyroid gland during CND. Devascularization, inadvertent removal, and parenchyma impairment are regarded as the major causes of hypoparathyroidism in thyroidectomy procedures [12]. In addition, compared with superior parathyroid glands, inferior parathyroid glands are more difficult to be localized and preserved in situ due to a more variable distribution [13].

Therefore, based on previous studies, we observed the distribution and nutritional vascular features of inferior parathyroid glands and developed a classification system for inferior parathyroid glands in total parathyroidectomy of patients treated in our institute with secondary or tertiary hyperparathyroidism due to chronic renal disease. Based on the classification system of inferior parathyroid glands, the new techniques including the designed dissection of the region adjacent to the lower pole of the thyroid and strategic protection of inferior parathyroid glands had developed in the CND procedure. The purpose of the current study is to access the protective effects of the techniques on inferior parathyroid gland protection.

## Methods

### Patients

In this retrospective study, a total of 197 PTC patients with total thyroidectomy and concomitant CND from January 2015 to June 2018, treated at the department of thyroid surgery, Second Affiliated Hospital of Nanjing Medical University, Nanjing China, were enrolled. Indication of total thyroidectomy was in line with the guidelines of the ATA for the management of differentiated thyroid cancer [14]. Prophylactic unilateral CND was routinely performed. Bilateral CND was carried out when the following conditions were met: bilateral PTC, overt lymph node metastasis in the central neck compartment, malignant focus located in the isthmus of the thyroid.

Patients who met the following criteria were excluded: previous neck surgery, concomitant parathyroid procedure, chronic renal diseases, severe dysfunction of the digestive system, abnormal serum parathyroid hormone (PTH), and calcium in a preoperative laboratory test. Eighty-nine patients whose operations were carried out with traditional meticulous fascia dissection before January 2017, were divided into group A. One hundred eight patients, who underwent active location and vascular preservation of inferior parathyroid gland from January 2015 to June 2018, were assigned as group B. In order to study the protective effect of the new techniques on inferior parathyroid glands (IPGs), 18 patients from group A who underwent auto-transplantation of IPGs were divided into the At group, and the 12 patients from group B who underwent auto-transplantation of IPGs were arranged into group Bt. Preoperative diagnosis of each patient was determined through fine needle aspirate biopsy guided by ultrasonography or two-dimensional ultrasonography alone.

Routine performance of prophylactic central neck dissection (CND) in patients with papillary thyroid carcinoma is controversial at present. It is recommended advisable by/in either Chinese or Japanese guidelines for the management of differentiated thyroid carcinoma [15, 16]. However, it is only recommended for patients with advanced disease (such as T3, T4 staging) in the corresponding guidelines of American Thyroid Association (ATA)

### The classification system of inferior parathyroid glands

- (1) *Type A*. It is the thyroid associated type, indicates IPGs that adhere to the surface of the lower thyroid pole, accounts for 53% of all IPGs, usually shares the same arterial branch of the inferior thyroid artery with the adjacent thyroid tissue. An IPG of type A is referred to as type A1 when its pedicle takes its origin from a prominent branch of the inferior thyroid artery, while other IPGs of type A are divided into type A2, whose pedicle usually arises from a slim branch of inferior thyroid artery or cannot be confirmed at all during the operation.
- (2) *Type B*. It is the thymus associated type, indicates IPGs that are located in the thymus or thyroid thymus ligament, and usually share the same branch of inferior thyroid artery with the ipsilateral thymus lobe.
- (3) *Type C*. It is the aberrant type, not closely related with the thyroid or thymus, can be found in various locations but is usually found in the tracheoesophageal groove, irrigated by a branch of the inferior thyroid artery to the thyroid, trachea, esophagus, or anterior neck muscles.

**Fig. 1:**
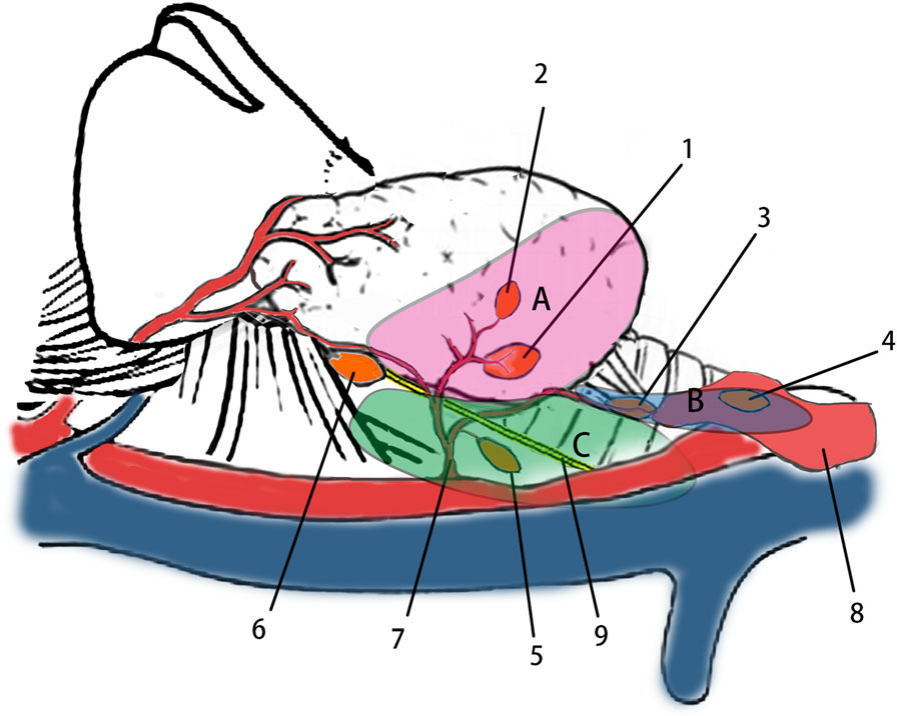
1. Inferior parathyroid gland (IPG) of type A1. 2. IPG of type A2. 3. IGP of type B1. 4. IPGSof type B2. 5. IPG of type C. 6. Superior parathyroid gland. 7. Inferior thyroid artery. 8. The tongue of thymus. 9. The recurrent laryngeal nerve. **a** Area refer to the back surface of inferior pole. **b** Area indicate the belt occupied by the tongue of thymus and thymus-thyroid ligament. **c** Area represent tracheoesophageal groove

The surface of the lower thyroid pole and the belt area occupied by the thyroid-thymus ligament are the most common habitats of inferior parathyroid glands, which are referred to as “one surface, one belt” in order to facilitate the clinical practitioner’s understanding of the classification system. The classification of inferior parathyroid glands is shown in Fig. 1.

### The designed dissection of the area adjacent to the lower pole of the thyroid and strategies for the protection of inferior parathyroid glands

The position and incision employed were the same as a normal thyroidectomy. Surgeons stood at the ipsilateral side of the involved lobes armed with × 2.5 loupe. The following approaches might be special for the techniques in the present study.

#### Developing surgical space

The potential space between the pre-tracheal fascia and the surrounding anatomical structures, such as the anterior neck muscles and the carotid sheath, were dissected adequately and deliberately to the lateral aspect of the esophagus. Special care should be taken for avoiding injury of recurrent laryngeal nerve (or no recurrent laryngeal nerve, occasionally), especially on the right.

#### Identification of key structure

The stem of the inferior thyroid artery was often exposed if it existed. The recurrent laryngeal nerves were usually identified at the intersection of the recurrent laryngeal nerve and the inferior thyroid artery from the lateral approach.

#### Exposure of surface of the lower pole of the thyroid, the tongue of the thymus, and the thymus-thyroid ligament

The pre-tracheal fascia was incised along the lateral edge of the neck thymus and the thymus-thyroid ligament, then the stem of the inferior thyroid artery. Lymph node and fat tissue of the central neck compartment were gently separated from the surface of the lower pole of thyroid, thymus, and thymus-thyroid ligament.

#### Identification of inferior parathyroid glands

The inferior parathyroid glands are identified at the surface of the lower thyroid pole and the belt area of the thymus-thyroid ligament and neck thymus. If inferior parathyroid glands were not found, thyroidectomy was performed with meticulous capsular dissection technique.

#### Strategies for preservation of inferior parathyroid glands

Parathyroid glands of A1 type were usually divided from the surface of the thyroid together with the nutrient vascular. A2 type inferior parathyroid glands are often divided from the thyroid surface together with a patch of thin thyroid tissue. When the dissection was completed, the blood circulation of the inferior parathyroid glands is assessed. Inferior parathyroid glands with suspected blood circulation obstacles are implanted in the sternocleidomastoid muscle after being cut into small pieces of 1 × 1mm^3^. B type inferior parathyroid glands were preserved in situ by freeing the neck thymus and thymus-thyroid ligament from the surrounding tissue. The branches of the inferior thyroid artery to the thymus are usually preserved carefully if they exist. Type C inferior parathyroid glands were identified during the central neck dissection stage. Preservation in situ or auto-transplantation of this type of inferior parathyroid glands depended on the discretion of the surgeon. After the inferior parathyroid glands were isolated, a thyroidectomy was performed using a meticulous capsular dissection technique. Superior parathyroid glands were identified and preserved in situ as far as possible.

#### Dissection of central neck lymph node

**Fig. 2:**
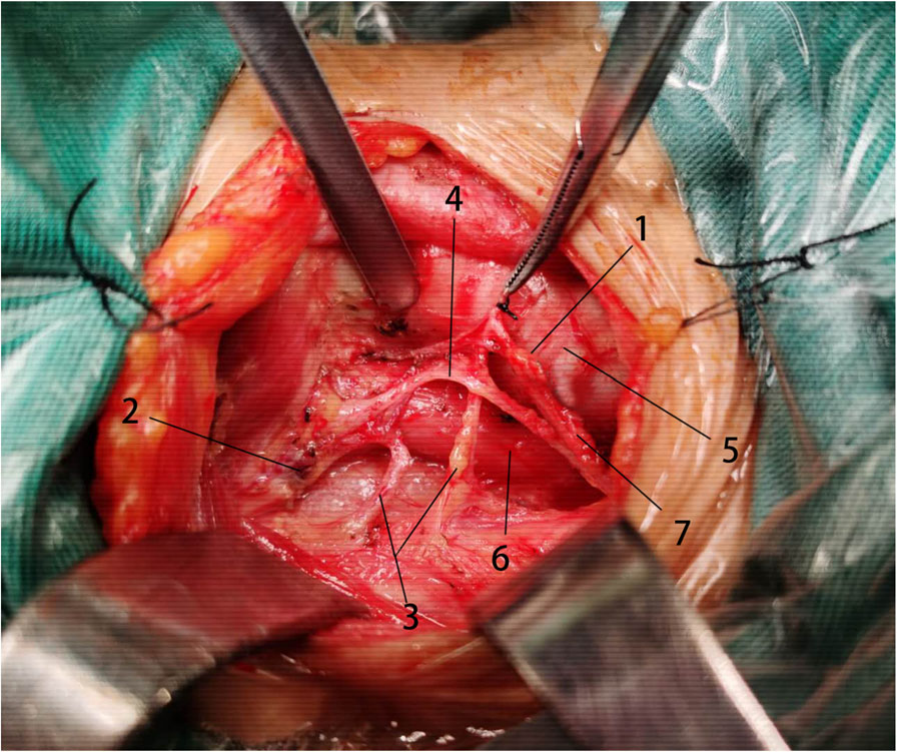
1. Inferior parathyroid gland. 2. Superior parathyroid gland. 3. Inferior thyroid artery. 4. Recurrent laryngeal nerve. 5. Trachea; 6. Esophagus. 7. Thyroid-thymus ligament

After the removal of thyroid glands, the major branch of the inferior thyroid artery and the recurrent laryngeal nerve were further skeletonized and preserved. Then, paratracheal and pretracheal lymph nodes were removed en bloc from the basin of the tracheoesophageal groove and the surface of trachea (Fig. 2). Prelaryngeal nodes were resected separately or together with the isthmus of the thyroid gland.

### Perioperative management and long-time follow up of hypocalcemia

Serum parathyroid hormone and total calcium were tested preoperatively and on the morning of the first postoperative day. A regime including 2 g calcium gluconate was routinely administrated to each patient through venous transfusion on the day of the operation and was continued for several days in patients with hypocalcemia symptoms. All patients with hypocalcemia symptoms were given oral vitamin D and carbonate for 2 weeks. The treatment was continued in patients with constant hypocalcemia symptoms to guarantee serum calcium above 2.0 mmol/L without hypo calcium symptoms. Serum parathyroid hormone and total calcium were determined at the following times: 1, 2, 3, 6, and 12 months after the operation.

### Clinical variables employed

In order to assess the protective effects of the techniques employed, the following clinical variables were adopted: serum parathyroid hormone and calcium on the morning of the first postoperative day; the incidence of chemical hypoparathyroidism, defined as serum parathyroid hormone below the low range of normal reference value (12-88 pg/ml) combined with hypocalcemia; the incidence of permanent hypoparathyroidism, defined as hypocalcemia combined with low or inappropriate serum parathyroid hormone level 1 year after the primary operation.

### Statistical analysis

Data were analyzed with IBM SPSS Statistics Version 24. Continuous parameters were presented as mean ± SD or median (quartiles). Two independent sample *t* tests or Mann-Whitney *U* tests were employed for the comparison of numeric variables. The Pearson chi-square test or Fisher’s exact test were adopted for categorical variables. Statistical significance was defined as *P* value < 0.05.

## Results

### General characteristics of the patients

A total of 197 patients who underwent a total thyroidectomy and concomitant central neck dissection were enrolled in the current study. The general characteristics of these patients are shown in Table 1. There are no significant differences between the two groups in the T stage, CND range, lymph nodes retrieved, and the ratio of IPTX. The ratio of metastasis is significantly lower in group A compared with group B (48.3% vs. 71.3%, *P* = 0.001). The ratio of AIPGS is significantly higher in group A compared with group B (20.2% vs. 10.2%, *P* = 0.048). The incidence of operation related complications except hypoparathyroidism were similar in two groups.

**Table 1:** General characteristics of the patients

		Groups		*P*
	A group (*n* = 89)		B group(*n* = 108)	
Age (years)	47.66 ± 12.10		43.85 ± 13.76	0.043^#^
Gender (female)	79 (88.8%)		75 (69.4%)	0.003^▲^
Hashimoto’s disease	18 (20.2%)		20 (18.5%)	0.763^▲^
Goiter	50 (56.2%)		57 (52.8%)	0.633^▲^
T stage				
T1	63 (70.8%)		65 (60.2%)	
T2	7 (7.9%)		14 (13.0%)	
T3	17 (19.1%)		23 (21.3%)	
T4	2 (2.2%)		6 (5.6%)	0.133*
CND range				
Unilateral CND	58 (65.2%)		61 (56.5%)	
Bilateral CND	31 (34.8%)		47 (43.5%)	0.243^▲^
Lymph nodes retrieved				
Unilateral CND	9.03 ± 4.701		10.67 ± 4.668	0.059^#^
Bilateral CND	14.55 ± 8.045		15.23 ± 7.035	0.692^#^
Ratio of metastasis	43 (48.3%)		77 (71.3%)	0.001^▲^
Ratio of IPTX	11 (12.4%)		8 (7.4%)	0.241^▲^
Ratio of AIPGS	18 (20.2%)		11 (10.2%)	0.048^▲^
Pre-PTH (pg/ml)	43.40 (34.90,58.75)		43.80 (33.83,60.35)	0.835
Pre-Ca (mmol/L)	2.31 (2.23,2.38)		2.32 (2.25,2.39)	0.424
Postoperative complications				
Hemorrhage	1 (1.1%)		2 (1.9%)	1.000^▲^
Incision infection	1 (1.1%)		1 (1.1%)	1.000^▲^
Transient RLN injury	2 (2.2%)		3 (2.8%)	1.000^▲^
Permanent RLN injury	1 (1.1%)		1 (0.9%)	1.000^▲^
Postoperative hospital stay	5.20 (± 3.28)		5.26 (± 2.08)	0.892^#^

T staging according to 8th edition TNM staging system of AJCC

*Ratio of metastasis* number of patients with lymph nodes metastasis/number of patients studied, *Ratio of IPTX* number of patients underwent inadvertent parathyroidectomy pathologically confirmed in specimens/number of patients studied, *Ratio of AIPTS* number of patients on who auto-transplantation of inferior parathyroid glands were carried out/number of patients studied, *Pre-PTH* preoperative PTH, *Pre-Ca* preoperative total calcium

*Mann-Whitney *U* test

^#^Student’s *t* test

^▲^Pearson chi-square test or Fisher’s exact test

### Comparison of clinical parameters between group A and group B

The perioperative serum parathyroid hormone and total calcium are shown in Table 2. The incidence of transient hypoparathyroidism sharply dropped in group B, compared with group A, from 32.6 (28/89) to 8.3% (9/108) (*P* < 0.001).

**Table 2:** Comparison of postoperative biochemical variables between group A and B

Biochemical variables	Groups			
	Group A (*n* = 89)	Group B (*n* = 108)	*Z*	*P*
Post-PTH (pg/ml)	18.80 (8.85, 28.50)	30.35 (21.25, 42.78)	5.238	< 0.001
Post-Ca (mmol/L)	2.00 (1.89, 2.12)	2.05 (1.97, 2.13)	2.339	0.019

Mann-Whitney *U* test was employed

*Post-PTH* postoperative PTH, *Post-Ca* postoperative total calcium

**Fig. 3:**
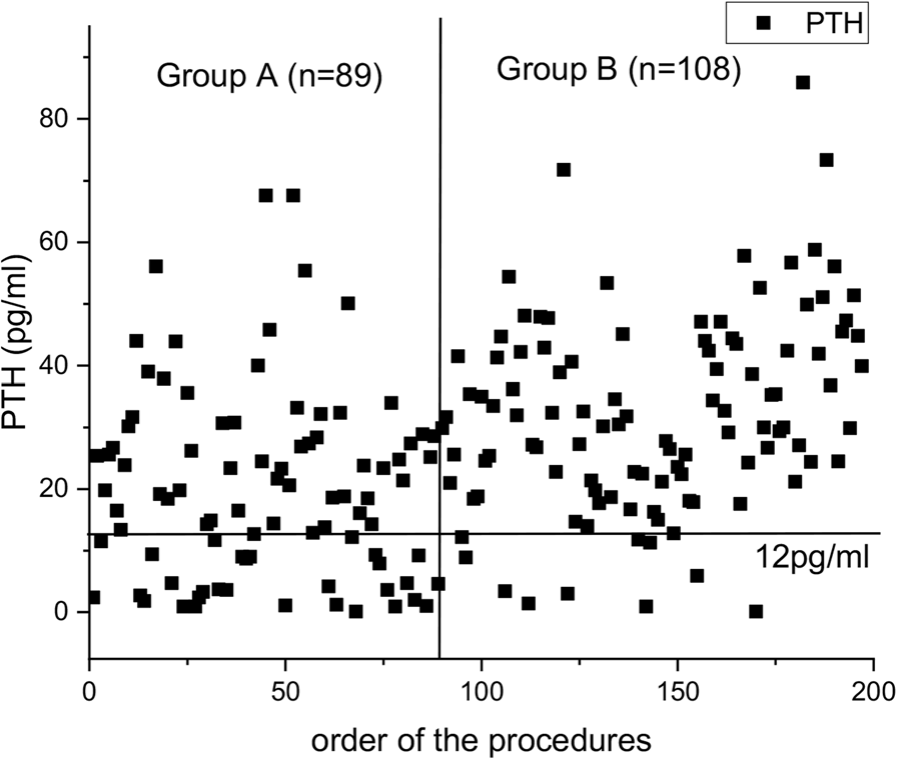
The serum parathyroid hormone level of the first operative day is demonstrated

A total of 170 patients in two groups had complete follow-up data. The incidence of permanent hypoparathyroidism significantly decreased in group B from 8.8 to 1.0% (*P* = 0.017). The serum parathyroid hormone level of the first operative day is demonstrated in Fig. 3, according to the order of the procedures performed.

### Comparison of clinical parameters between group At and group Bt

The serum parathyroid hormone and total calcium on the first postoperative day are demonstrated in Table 3. The incidence of transient hypoparathyroidism was not significantly different between group At and group Bt, 33.3% (6/18) VS 18.2% (2/11) (*P* = 0.671). A total of 24 patients in group At and group Bt had complete follow-up dates. The incidence of permanent hypoparathyroidism is not significantly different between group At and group Bt, 7.7% (1/13) VS 0.0% (0/11) (*P* = 1.000).

**Table 3:** Comparison of biochemical variables between group At and Bt

Biochemical variables	Groups			
	Group A (*n* = 18)	Group B (*n* = 11)	*Z*	*P*
Pre-PTH (pg/ml)	45.00 (35.43, 58.13)	41.40 (31.60, 59.80)	0.652	0.515
Post-PTH (pg/ml)	15.40 (6.85, 24.58)	18.40 (14.70, 24.30)	0.472	0.637
Pre-Ca (mmol/L)	2.31 (2.24, 2.38)	2.37 (2.29, 2.41)	0.856	0.392
Post-Ca (mmol/L)	1.97 (1.82, 2.05)	1.99 (1.89, 2.05)	0.562	0.574

Mann-Whitney U test was employed

*Pre-PTH* preoperative PTH, *Post-PTH* postoperative PTH, *Pre-Ca* preoperative total calcium, *Post-Ca* postoperative total calcium

## Discussion

The routine performance of prophylactic CND can cause hypoparathyroidism, so patients who underwent total thyroidectomy and concomitant bilateral central neck dissection can develop permanent hypoparathyroidism. Therefore, it is worth exploration about how to protect the parathyroid gland during CND. Devascularization, inadvertent removal, and parenchyma impairment are the major causes of hypoparathyroidism in thyroidectomy procedures; however, it is very difficult to locate inferior parathyroid glands and then to preserve them due to a more variable distribution, and there are no best methods now. Therefore, we intended to observe the distribution and nutritional vascular features of inferior parathyroid glands and developed a classification system for inferior parathyroid glands in total parathyroidectomy.

It is always a vital issue for thyroid surgeons to find the best way to protect the parathyroid during a thyroidectomy. Several techniques have been developed for the intraoperative protection of the parathyroid glands in recent years. It has long been proved that the blood supply of the parathyroid came from a tertiary branch of the inferior thyroid artery and advocated ligation of the inferior thyroid artery should be performed at the surface of the thyroid to avoid impairing the blood supply to the parathyroid, which is the first description of the capsular technique [17]. Zhu and his colleagues had divided the parathyroid gland into A and B types according to the anatomic relationship between the parathyroid gland and the thyroid [18]. Type A is a compact type, meaning that this type of parathyroid gland closely connects with the thyroid, and thus is relatively difficult to be preserved in situ in operation. Type B is a no-compact type, meaning that a natural gap exists between the thyroid and the parathyroid glands. Thus, this type of parathyroid gland is often easier to be preserved in situ than those in Type A. In another study, it was also shown that the auto-transplantation of the inferior parathyroid gland prevented permanent hypoparathyroidism and decreased the incidence of lymph node recurrence of the central neck compartment [19]. In the current study, the inferior parathyroid gland was classified according to the anatomic relationship between the thyroid and thymus, and further categorization of the type A inferior parathyroid gland was based on the features of the blood supply of the inferior parathyroid gland. This classification method can assist the surgeon in deciding whether in situ preservation or auto-transplantation should be performed to protect the function of the inferior parathyroid gland. We also found that elaborately preserving the parathyroid gland vasculature is key to the protection of the parathyroid gland and the significantly decreased incidence of both transient and permanent hypoparathyroidism. This was in line with the study of Park, which indicates that the function of the parathyroid gland is effectively protected by the preservation of the parathyroid gland vasculature [20]. In addition, Wang had introduced the concept of the lay of the thymus-blood vessel- inferior parathyroid gland and demonstrated the protective effectiveness of properly preserving the structure in CND, which is similar to the method adopted for the parathyroid gland of type B in our current study [13]. Cui, et al. also introduced a classification system of the parathyroid gland based on the relationship between the blood supply of the parathyroid gland and the thyroid [21]. According to their classification system, when the blood supply of the parathyroid gland does not depend on the thyroid, it is categorized as type A; when the blood supply of the parathyroid gland partly depends on the thyroid, it is categorized as type B; when the blood supply of the parathyroid gland completely depends on the thyroid, it is categorized as type C. So, similarly, in the current study, the parathyroid gland was preserved in situ or auto-transplanted during thyroidectomy based on the relationship between its blood supply and the thyroid.

The techniques adopted in the current study effectively preserved the function of the inferior thyroid glands in the CND procedure, and, as a result, serum parathyroid hormone and total calcium on the first operative day significantly increased. Additionally, the incidence of chemical hypoparathyroidism dropped sharply, which was proven to be closely associated with symptomatic hypocalcemia and permanent hypoparathyroidism [22–24]. On the other hand, when the inferior parathyroid glands were auto-transplanted, although usually only one inferior parathyroid gland was involved, there were no significant differences between the clinical parameters of the At and the Bt group. Therefore, with the new techniques, the improvement in the function of the parathyroid contributed to the protection of the inferior parathyroid glands. In recent years, there was some controversy around the treatment of the inferior parathyroid glands in the procedures of thyroidectomy. Zhu and his colleagues advocated that inferior parathyroid glands should be transplanted in the central neck dissection procedure in order to decrease the possibility of permanent hypoparathyroidism and the recurrence of lymph nodes in the central neck compartment [25]. However, the study by Lonpch showed that auto-transplantation not only cannot prevent patients from permanent hypoparathyroidism but it also increases the incidence of transient hypoparathyroidism [26]. The results of the current study show that the majority of inferior parathyroid glands can be safely preserved in situ. Auto-transplantation can be employed as a salvage treatment when the blood supply of the inferior parathyroid glands is impaired.

The new techniques protected the function of the inferior thyroid glands by locating the inferior parathyroid glands and preserving their blood supply during the central neck dissection procedure. In the 1970s, Thompson delineated the meticulous capsular dissection technique by describing the development of a plane between the thyroid capsular and the inferior thyroid artery to avoid the impairment of the vessels of the parathyroid glands [27]. At present, the meticulous capsular dissection technique is comprehensively accepted as a crucial method to protect the functions of parathyroid glands in the procedure of thyroidectomy. However, when CND and thyroidectomy are simultaneously performed, active location and protection of the vessels of the parathyroid glands become more necessary because the parathyroid glands and their blood supply are at a higher risk of impairment during the procedure. The situation is more serious for the inferior parathyroid glands due to their variable locations. The new classification system of inferior parathyroid glands demonstrated that the majority of inferior parathyroids are situated at the back surface of the lower pole of the thyroid and the belt area occupied by thyroid-thymus ligament and the upper horn of the thymus. The concept of one surface and one belt reflects the regularity of inferior parathyroid gland distribution, in line with the anatomic studies [28], and provides the anatomic basis of the technique called as the designed dissection of the area adjacent to the lower pole of the thyroid. Inferior parathyroid glands are routinely located and identified through this technique. The strategies of inferior parathyroid gland protection provide practical references for decision-making of whether a parathyroid gland can be preserved in situ. Parathyroid glands of A1 and B types, generally accepting blood supply from a sturdy branch of the inferior thyroid artery, can be safely preserved in situ in most of the cases, through dissecting the plane between the inferior thyroid artery and the thyroid using the meticulous capsule dissection technique as described by Thompson. However, with parathyroid glands of type A2, a patch of thyroid tissue between the parathyroid glands and the major branch of the inferior thyroid artery is usually dissected together with the inferior parathyroid glands when the oncologic safety is guaranteed. Otherwise, it should be auto-transplanted without hesitation. The blood supply status of the inferior parathyroid gland should be assessed deliberately when it is completely divided from thyroid. Active auto-transplantation should be performed in a situation where the blood supply of the inferior parathyroid gland is noticeably damaged. Additionally, it is also very crucial to preserve the venous drainage of the inferior parathyroid gland. The study of Lee demonstrated that preserving the inferior thyroid vein benefited in protecting the function of the parathyroid glands [29]. In the current study, the inferior thyroid vein or fibrous connective tissue between the inferior parathyroid and trachea is individually preserved according to the relative anatomic position of the inferior parathyroid gland and the inferior thyroid vein.

## Conclusions

Due to variable location and close anatomic relationship with lymph nodes, fat, and connective tissue in the central neck compartment, it is a challenge for surgeons to protect the inferior parathyroid gland during a thyroidectomy procedure and central neck dissection. The new classification system reflects the feature of the blood supply of the inferior parathyroid gland and the relative anatomic relationship between the parathyroid gland and the thyroid, and thymus and ligament of the thymus-thyroid. The designed dissection of the area adjacent to the lower pole of the thyroid and the strategies for inferior parathyroid protection based on the new classification of inferior parathyroid gland improved the maneuverability of IPGs protection in the procedure of thyroidectomy and central neck dissection.

## Data Availability

We declared that materials described in the manuscript, including all relevant raw data, will be freely available to any scientist wishing to use them for non-commercial purposes, without breaching participant confidentiality.

## Abbreviations

PTC: Papillary thyroid carcinoma
CND: Central neck dissection
ATA: American Thyroid Association
PTH: Parathyroid hormone
IPGs: Inferior parathyroid glands
